# Odontological Society of Pennsylvania

**Published:** 1894-01

**Authors:** 


### ODONTOLOGICAL SOCIETY OF PENNSYLVANIA.

THE regular monthly meeting of this Society was held at 1228 Walnut Street, Philadelphia, November 11, 1893, the President, Dr. E. T. Darby, in the chair.

The essayist of the evening, Dr. Bonwill, not having arrived at the conclusion of routine business, the President suggested that subject No. 10 of the series of questions be taken up,—"The Etiology of Pus Formation,"—and called on Dr. Truman to open the discussion.

### DISCUSSION.

*Dr. Truman.*—The etiology of pus formation is by no means a settled question. The Waller-Conheim theory of the emigration of the white corpuscles of the blood is met by the school of Virchow, Von Recklinghausen, Stricker, etc., with the older theory, enunciated by the first named,—that of the retrograde metamorphosis of tissue,—and while the opinion of histologists leans towards the emigration theory, there is sufficient evidence to support the assertion that the origin of pus may be based on both processes.

The more recent and perhaps quite as important subject in this connection is the effect of pathogenic germs in the production of pus. It has been asserted that the formation of this is impossible without their active co-operation. To demonstrate their presence in all centres of suppuration has been found surrounded with difficulty; in fact, there is ample evidence to prove that abscesses occur in which no germs are present.

It has been demonstrated by Miller and others that the air is not a very serious source of infection; and that it is not probable that micro-organisms reach the focus of inflammation through that medium in any considerable quantity. If this be true, it seems to leave but two sources of infection, direct contact with septic matter and through the circulation. While the possibility of centres of inflammation being produced through the blood needs no substantiation, it is equally true to say that all suppuration has not its origin from this source, or that abscesses are always produced through the blood. The results described by Peyrot, Kartoulis, and Laveran, in abscess of the liver, showing the absolute sterility of pus, indicates that at least the living organism is not always necessary in pus production. This is in a measure confirmed by Steinhaus, that ptomaines are "capable of producing suppuration without living organism." Janowski, Lemaire, Rosenbach, Grawitz and De Bary, and others have shown that chemical irritants will produce suppuration without the aid of micro-organisms. While this is disputed by others, their assertions, founded on experimental researches, have not been disproved.

It is probable that at the later stages of pus formation micro-organisms become active factors in its production. The minute lakes of pus found in pulps in a state of inflammation—pulpitis—must have their origin in immigration of the leucocytes, and cannot, I think, be ascribed to any retrograde process. To what extent this may be considered as the action of pyogenic germs has yet to be determined.

*Dr. Brubaker.*—In the formation of pus, the first element, apparently, has been overlooked. The formation of pus is, of course, simply one of the stages of inflammation, and inflammation is invariably preceded by a state of congestion, by which is meant a condition of paralysis of the walls of the smaller blood-vessels. As soon as there is this impaired circulation there is an aggregation of the white blood-corpuscles along the walls and the emigration into the surrounding tissue. With the cessation of the blood-flow there is at once an impairment in the nutrition of these tissues which leads to a lessening of their vitality. This is the first step, and which has been alluded to as retrograde change in the tissue-cells. The mere emigration of the white blood-corpuscles through the cells of the blood-vessels, carrying with them various germs, would not in themselves give rise to the formation of pus. Germs in and of themselves cannot produce pus until there is impaired vitality of the tissues, which allows them to disintegrate the tissue molecules. Therefore I don't see how it is possible for pus to be formed anywhere in the body, except on the surface, unless you have the two factors, impaired vitality and presence of the germ. How the germ gets in the subcutaneous tissue to produce pus is, of course, a question, but I think they are absorbed by the white blood-corpuscles and are carried by them to the tissues, because they are constantly migrating.

At this point Dr. Bonwill arrived and read his paper on hypnotism.

(For Dr. Bonwill's paper, see page 21.)

DISCUSSION.

*Dr. Faught.*—I am sure we are all very much interested in this paper which Dr. Bonwill has read, but I feel quite confident he has only entered upon the very outside of the subject of hypnotism. Thirty-four pages would hardly cover a full treatment of the subject. I am quite disappointed in that he has only gone into its primary stage, that every one who has given any thought to the subject is familiar with. The concentration of thought in the patient, his idea of quietness and silence,—these things we find in almost every article written upon the subject.

I have written a treatment of one thought in connection with the subject, and if I clearly get you to see that one thought I shall have started you, I think, on the right road to the understanding of hypnotic influence.

The study of hypnotism, or of hypnotic suggestion, though fitfully pursued for many years, has only in the last two or three made any pretence to classification as a science. It is now being classed under the medical sciences. They, however, are positive in character, while it by nature is occult. In speaking, therefore, of such abstruse matters, there is often apparent disagreement when in reality the opinions advanced are in perfect harmony. This apparent disagreement is due to many causes, but the main reason of difference has at all times arisen from the attempt to define what in point of fact is undefinable, and is necessarily the outcome of the use of terms which are not susceptible of accurate definition. Hypnotism, being allied to spiritistic phenomena, requires that each student of it must for himself be the supreme judge of whether what is conveyed is or is not; for the principles governing the latter are involved in a primary study of the former. Everything proceeding from the invisible world has always come through human instrumentality, and has differed according to the conditions of the transmitting medium and those of the persons to whom directed. All such communications are therefore imperfect, and relative in value. Nevertheless, the unseen world is teeming with intelligences whose action upon this one is very direct, and is governed by laws, most of which are hidden from us; and those which are known are not comprehensible to the many.

With these introductory remarks and premises, I propose to confine myself to one point of study. I wish to explain to-night, if possible, how the hypnotic influence is conveyed from one person to another, apparently separated from each other. I wish to show that the separation is not as complete as appears to the observers, but that the individuals are in physical contact. For the purpose of this demonstration it is to be realized that our external senses are not tests upon which we can rely for anything, being organs for the transmission of sensations. It is an irrefutable axiom, that a thing is to the sense which perceives it what to that sense it seems to be. No one will deny that when they lay their hand upon a table or desk that there is actually no contact. What is true in the larger field is true in the lesser. No atom of matter in the hand is in contact with another atom, for each atom of matter everywhere is surrounded by what is called its "dynasphere,"—that is, a definite invisible circle manifesting perturbations derived from the energy of the atom. What is the constitution of this dynasphere can only now be inferred to be matter in still more minute atoms, even so finely divided as to be unseen by any known means. Their influence is felt though unseen. We are accustomed to this condition in every-day life, for science admits matter exists in gases and ether, in light, and in heat; indeed, nothing has yet been discovered of which it can be asserted that no matter is there. It must, therefore, be in the atomic dynaspheres themselves, and it is evident that we can conceive of no limit to it. The dynaspheres of atoms are in their turn surrounded by dynaspheres, and so on *ad infinitum*. This dynaspheric force is the agent of the phenomena of hypnotism, spiritism, and occultism generally. This physical thing is nothing more or less than what we have been in the habit of calling "spirit" when we wished to separate it from what is termed "ordinary matter." It energizes and does work in and upon "ordinary matter," and is a separate form of matter, infinitely refined and infinitely rapid in its vibrations. This dynaspheric force it is which, passing from the organism of the operator by a continuous chain of matter in harmonious vibration, impinges upon the organism of the hypnotized patient and controls his will, his acts, and words. Here we have the secret of the attraction or repulsion we call love or hate, sympathy and antipathy, etc., which we produce upon our neighbors or they produce upon us. We but express the truth when we commonly say that we make or receive an impression. Scientifically speaking, all individuals in every-day life more or less hypnotize each other. To understand hypnotism we are therefore to clearly grasp the idea that physical, mental, moral, and emotional forces are all material. In this concept is a field of undreamt-of potency.

*Professor Newbold.*—I think I can hardly say anything directly relevant to the statements of the last speaker. It must be very evident to all of you that the theory which Dr. Faught has advanced involves not merely the phenomena of hypnotic suggestion, so called, but practically, we might say, the complete philosophy of life, here and hereafter as well. It seems to me also that the theory which he has advanced,—the spiritistic theory,—as an explanation of the phenomena of hypnotism, is possibly a little premature. I would not say that the facts we have are entirely inconsistent with it, but I would say that those who have most carefully studied hypnotism, and those who at present are best recognized as authorities, are not inclined to assume such an explanation. I don't like to use the word hypnotic force, as a force over and above the ordinarily recognized forces of physics and chemistry.

As to the points raised by Dr. Bonwill, it appears to me that the facts he presents all would be inclined to accept. It seems to me also, however, that we might differ with him very decidedly in the strong line of demarcation which he is inclined to draw between hypnotism as such and ordinary sleep and the phenomena of suggestion as distinguished from hypnotism, which he seems inclined to attribute to some personal magnetism proceeding from the operator. Without denying the existence of such magnetism, I think it is evident we must go as far as possible with the recognized facts and ordinary principles of psychology before we need to introduce any such new or hypothetical influence.

I do not deny that there are certain facts which are very well distinguished, which do indicate the necessity of assuming something analogous to thought induction, the possibility of one mind affecting another mind by some means other than the recognized forms of sense, but I think one would prefer for a long time to refrain from formulating any defined theories to account for such facts. I think, first, we need careful, competent, and trained observers ; very few are either careful, competent, or trained. We need to get the facts and place them beyond dispute. At present they are disputed by men for whose opinion we must have very great regard. Consequently you might say I stand to-night in a more or less sceptical position in relation to the theories and in a cautious position in relation to facts.

*Dr. Truman.*—We have Dr. Warren with us this evening. He has had considerable experience with hypnotism, and I should be glad to hear from him upon the subject.

*Dr. Warren.*—I have been more or less familiar with the phenomena of hypnotism all my active life. My father used it to some degree in his practice of medicine many years ago. Of late years I have employed it to a limited extent in my own practice. I would say with Dr. Faught, that I am disappointed in the paper presented this evening; I came with the expectation of hearing something more advanced,—something of a scientific nature presented to a body like this. While as a guest of the Society I appreciate the courtesy of the floor, I am frank to say that the essay was so elementary that I must decline to discuss it.

I will say this, however, of hypnotism, that I believe we can use it in our practice to a remarkable degree. I believe, too, that there is a great deal in the operator himself, and there is something more in it than suggestion; in fact, from personal experience, I have more than once laid the patients or subjects upon their backs in a profound sleep without speaking a word,—a mental suggestion, if you please. In practice, when a new patient presents himself or herself, I always endeavor to reach the mind and act upon it before operating upon the teeth. I think if we would all take that time with our patients to secure their confidence and then retain it, we would meet with more success in practice and help remove the fear and dread of the dental chair.

I might speak of one case occurring recently. A child—a young girl—who had never been in a dentist's chair was brought to me. While preparing for the operation and washing my hands I found the little one was crying, and the mother trying to get her to step up into the chair herself, or endeavoring to force her to do so. I asked the mother to step into the other room and make herself comfortable and leave the child to me. In a moment or two I had the little one's confidence, and proceeded to perform the operation, which was the insertion of three fillings, without a flinch or complaint from the child, the work being thoroughly done. She told her mother that it was better than playing dolls; then seeing that the remark was rather amusing to us, she turned about and explained why she said it, which was that she was entirely at ease in the chair, and was interested in the work, but when she was playing house with the other children they had their troubles and quarrels.

That is simply one case, and shows that with a little care we can make the dental chair really comfortable.

*Dr. Truman.*—This subject is a profound one, and has claimed the attention of some of the best thinkers of the world.

From my earliest boyhood I have been interested in this and kindred topics, and this has not lessened as it has become a recognized subject for scientific investigation. It has, in all ages of the world's history, been the custom to repudiate unknown and startling statements, no matter however well supported, until they have received endorsement from recognized scientific bodies. Now, I believe that truth can originate from very lowly sources; in fact, I may go farther and say that the majority of truths that have eventually come to be recognized as such have had their origin in humble places, and not from the higher scientific circles of the period.

The prevailing idea that animal magnetism, mesmerism, hypnotism, for they are all synonymous terms, had their origin in the work of Frederick Anton Mesmer is an error. It was hoary with age before Mesmer was born. The other generally received opinion that Mesmer was a charlatan is equally erroneous. Those who are familiar with his history cannot truthfully denominate him as a quack. It is true he practised methods which at this day would be regarded as indefensible by correct thinkers, but in judging him the peculiar conditions of the period in which he worked must be taken into consideration, and also the fact that he really believed the modes adopted were necessary to the production of the magnetic sleep. The credit due to Mesmer, and it should be given him in full measure, was that he proved the existence of this power. The practical knowledge he possessed was doubtless received from Gassner, a priest of Switzerland, who effected cures by manipulation. Another idea in regard to Mesmer, that he was an uneducated man, is disproved by the fact that he graduated in medicine in Vienna, and for some time practised his profession.

Reichenbach fifty years ago demonstrated, after strictly scientific work continuing over five years, the existence of a force in the human organism, which he named odyle. This odylic force or emanation could be observed in sensitive subjects. He did not attempt to give any explanation as to the origin of this, but if the result of his experiments can be accepted, he proved its presence in the human subject. When Professor Newbold claims that until we have exhausted all sources of explanation we have no right to assume the existence of a force analogous to electricity, he is not, I think, standing on tenable ground. The statement assumes that there has been no evidence adduced in this direction worthy of consideration. Has he made himself familiar with all the work of the past? I have no question but this has been done thoroughly with the means at command, but there are sources of information not always within the reach of those willing to work, and until these be sounded it is perhaps proper to take a non-committal position. The entire subject of occultism, in its practical and theoretical bearings, must be cultivated to understand this question in its general relations.

Personally my experience and study of the phenomena connected with hypnotism go back a half-century. It was somewhat of a family affair, as my father, in his medical practice, made much use of it in minor surgery, as the extraction of teeth.

During all this long period the mere idea of suggestion was not considered of vital importance. This was introduced in 1841 by James Braid, a surgeon of England, who denominated it hypnotism, or, more strictly, neuro-hypnotism, dropping the neuro subsequently. It was generally regarded as an unknown force inherent in the animal organism. Unless we accept Reichenbach's investigations, this has never been proved, but the evidence has been growing steadily through many decades that no other explanation will accord with the facts. I think the majority familiar practically with hypnotism will agree with me in the assertion that when passes are made from one individual to another, both the recipient and operator are conscious of an influence abnormal in character. I have personally felt this in marked degree traversing with electrical rapidity through the nerves, and at times producing almost local paralysis. This is not the result of suggestion. While it is true that mind can act readily on mind under proper conditions, I question its power as described by writers. I have repeatedly put persons to sleep, when in a highly nervous state, by making passes over the body and without touching the person. In one instance, in low, nervous fever, sleep was induced always by this process. This may be called suggestion; I prefer to regard it as induction of a current of unknown properties, and producing an equilibrium in the disturbed nervous force of another.

Many experiments not necessary to mention here have demonstrated the existence of a force in the animal economy not as yet recognized by science. That the needle of the compass has been moved repeatedly without contact is a well-attested fact. The most recent, and, perhaps, as well supported as any other of this class of experiments, is that of Dr. Julian Ochorowicz, with a sensitive, in Italy, in which the needle made movements, hither and thither, of fifteen degrees, by simply bringing the hand of the sensitive near, but not touching, it. The galvanometer was not in the least effected. The theory of suggestion will not explain this phenomenon. The fact that the galvanometer was not disturbed disproves the electrical theory.

These and similar experiments, extended from the time of Reichenbach, have, I think, shown at least the possibility of a force differing from any known power.

There is yet much to be discovered; indeed, the little we do know is simply the outermost fringe of the psychical problems yet waiting solution.

*Professor Newbold.*—I desire to say a word or two in reference to my previous remarks, which Dr. Truman has probably not understood. I do not deny the existence of such a thing as any method of communication between two minds by means other than those ordinarily recognized, but I have never seen such facts as would point in that direction. I think it unfortunate to invoke any hypothesis to explain facts which can be explained by laws of phenomena which we do not understand. A very large part of the phenomena of suggestion and hypnotism, taking the two together, can be explained by perfectly well understood laws, or, if not perfectly well understood, at least prevailing principles and general laws. If there be facts which cannot be explained by well-known hypotheses, it will be time enough then to bring in others.

*Dr. Gilbert.*—The subject is of great interest to me, and my success in practice is due to the use of hypnotism, more especially with children, although with grown persons to a considerable extent. I have gone so far as to extract teeth without pain, the patient stating that to be the case. I now call to mind a case in which I extracted a molar where I told the patient that it would not be felt, and no pain would be incurred, and the patient said after the extraction that no pain had been suffered, although it was strongly and well fixed in the jaw.

As to the personal electricity, I find sometimes in operating that it proves very uncomfortable and annoying; so much so that the soles of my shoes would require dampening to avoid affecting the patient while undergoing treatment; they have expressed it as though a pin had been stuck into them. That, of course, was electricity.

*Dr. Bonwill.*—If there is any magic in hypnotism then there is magic in rapid respiration. Every one knows it to be a physiological fact that the amount of air passing into the lungs will produce an obtunding effect. I have learned it in experiments upon myself. You can destroy the sensibility to pain, but increase the sensibility to touch; experimenting on myself with chloroform led me to that discovery. I say to my patients, "Breathe rapidly; breathe as hard as you can; this will not destroy your sensibility to touch; you will see me and feel me, and it will be exaggerated."

I urge my patients to breathe very rapidly, at the same time talking to them and making suggestions. While hypnotism will affect only about forty per cent. of cases, rapid respiration will affect all. The suggestions and addresses should be in a loud and commanding tone, they must be markedly affected by a positive voice, as a general commands his army.

*Dr. Faught.*—I think Dr. Bonwill makes a mistake in regard to the commanding tones and loudness of speech. My idea of command is the power back of what is said, and not the way in which it is said. A command used in the most quiet tone would bring about obedience when it is known the power exists back of it to enforce it. It is not the law but the power behind the law which gives it the effect.

*Dr. Warren.*—I wish to endorse what Dr. Faught has just said. The suggestion need *not* be made in the form of a loud command, but on the contrary can be made in a very quiet tone. I have often sealed the eyes of subjects so that it was impossible for them to open them, simply by saying, quietly, "You cannot open them." Sometimes we will find among our subjects a nervous young girl, who, when she finds it to be impossible to open her eyes, will commence to struggle, and, if allowed, would go into hysterics; but by saying, in the same quiet manner, "Stop struggling and your eyes will come open," she will stop and the eyes open at once.

The element of danger has not been brought out this evening. I would like to say a word in regard to that. From my experience and observations I believe there is no danger whatever except what the operator ignorantly or foolishly makes of it, by giving suggestions that are dangerous. If the operator is an intelligent man or woman, always respecting the subject's feelings, there is no danger or unpleasantness whatever. In nearly all cases the subject must be entirely passive and willing; then you can control them very easily. Operators should always have special training and understand thoroughly how to handle the subjects when they are in the hypnotic state.

Dr. McQuillan proposed Dr. Theodore F. Chupein for honorary membership, and the meeting then adjourned.

